# Artificial Intelligence-Based Bolt Loosening Diagnosis Using Deep Learning Algorithms for Laser Ultrasonic Wave Propagation Data

**DOI:** 10.3390/s20185329

**Published:** 2020-09-17

**Authors:** Dai Quoc Tran, Ju-Won Kim, Kassahun Demissie Tola, Wonkyu Kim, Seunghee Park

**Affiliations:** 1 Department of Civil, Architecture and Environmental System Engineering, Sungkyunkwan University, Suwon 16419, Korea; daitran@skku.edu (D.Q.T.); kastolla@skku.edu (K.D.T.); 2 Department of Safety Engineering, Dongguk University-Gyeongju, Gyeongju 38066, Korea; 3 School of Civil, Architectural Engineering and Landscape Architecture, Sungkyunkwan University, Suwon 16419, Korea; kwk1104@gmail.com; 4 Technical Research Center, Smart Inside Co., Ltd., Suwon 16419, Korea

**Keywords:** acoustic emission, digital signal processing, laser applications, machine learning, structural shapes, waves

## Abstract

The application of deep learning (DL) algorithms to non-destructive evaluation (NDE) is now becoming one of the most attractive topics in this field. As a contribution to such research, this study aims to investigate the application of DL algorithms for detecting and estimating the looseness in bolted joints using a laser ultrasonic technique. This research was conducted based on a hypothesis regarding the relationship between the true contact area of the bolt head-plate and the guided wave energy lost while the ultrasonic waves pass through it. First, a Q-switched Nd:YAG pulsed laser and an acoustic emission sensor were used as exciting and sensing ultrasonic signals, respectively. Then, a 3D full-field ultrasonic data set was created using an ultrasonic wave propagation imaging (UWPI) process, after which several signal processing techniques were applied to generate the processed data. By using a deep convolutional neural network (DCNN) with a VGG-like architecture based regression model, the estimated error was calculated to compare the performance of a DCNN on different processed data set. The proposed approach was also compared with a K-nearest neighbor, support vector regression, and deep artificial neural network for regression to demonstrate its robustness. Consequently, it was found that the proposed approach shows potential for the incorporation of laser-generated ultrasound and DL algorithms. In addition, the signal processing technique has been shown to have an important impact on the DL performance for automatic looseness estimation.

## 1. Introduction

As one of the most well-known connection methods, bolt connections are widely used for connecting components to structures because of their simplicity of maintenance disassembly. However, bolted joints may operate under various conditions during service life, such as humidity, high temperature and cyclic loads, which may reduce the preload of the bolts, cause structural instability, or even contribute to a catastrophic accident and failure of the entire structure [1]. Thus, effective monitoring and diagnosis of the bolt connections are necessary to ensure that structures are safe and reliable.

Although many academic works have explored different methods of detecting bolt loosening, including vibration-based measurements [2,3], electro-mechanical impedance methods [4,5,6,7,8], electrical conductivity techniques [9], ultrasonic-based measurements [10,11,12,13,14,15,16,17,18,19,20], and vision-based methods [21,22,23,24,25,26], much less research has been done on the applications of machine learning (ML) or deep learning (DL) algorithms in this field. Recently, ML and DL have become the breakthrough tools, particularly in the field of computer vision, to overcome the limitations of conventional structural health monitoring (SHM) and non-destructive evaluation (NDE). As introduced in a review study by Azimi et al. [27], ML and DL algorithms can be successfully applied to a wide range of applications in SHM, such as cracks, corrosion detection and structural component recognition [28,29]. Recent studies utilizing vision-based techniques with ML and DL algorithms for bolt looseness detection have been conducted [21,24,25,30]. These studies can be summarized as follows. In [21], the authors proposed a vision-based method incorporating images of bolted joints taken with a smartphone camera, and ML algorithms to detect the loosened bolts. Their approach was successful in detecting distinctively loosened bolts. In addition, using a similar approach as [21], in [24,25] the authors reported a DL algorithm approach with camera images to detect looseness in the bolt. However, with these techniques, the author can only detect whether the bolt is loose. Moreover, the level of looseness of the bolts was evident to the naked eye. These techniques cannot provide the torque value associated with the target bolt. In [30], the authors reported a new vision-based method for detecting the looseness of bolted joints in a flange connection. Nevertheless, the authors pointed out in the conclusion that the proposed method cannot quantify the level of the loosened bolts. From these state-of-the-art studies, the quantification problem in bolt looseness still remains a challenge and is an open field of research in terms of improvement. For this reason, our proposed method quantifies the looseness level with the aid of signals received from a laser ultrasonic system. Furthermore, our approach is based on a data-driven technique, i.e., a DL regression model with hundreds of thousands of trainable parameters, which can learn from thousands of samples and with the aid of a cross validation technique. These processes can overcome the limitations of conventional signal processing techniques, including the issues of noise cancellation and parameter sensitivity. Those methods mentioned above can be validated by observing how successful our trained model is, when tested on unseen testing data during the validation step.

Ultrasonic wavefield images can be created using Nd:YAG pulsed laser-based ultrasonic wave propagation imaging (UWPI). This system is composed of exciting and sensing units. A non-contact exciting unit consists of a pulsed laser [31,32], and the sensing unit can be a contact-type sensor [33,34,35,36,37] or a non-contact-type sensor such as a laser Doppler vibrometer [38,39]. This technique has various advantages over conventional contact-type transducers [40]. In addition to accelerating the scanning time, which is one of the disadvantages of UWPI, researchers [41] have presented a novel approach to solve this problem by using compressed sensing and DL algorithms. With these advantages, UWPI has become a promising tool in both NDE and structural health monitoring (SHM) [42]. Some studies have already applied wavefield imaging for detecting loose bolts [43,44,45]. Furthermore, in 2014, An et al. [46] developed a laser non-contact sensing and wireless excitation of piezoelectric transducers for detecting hidden damages in a steel box girder bridge. This research also showed the applicability of UWPI in the field. In 2018, Ye et al. [47] proposed a method for ultrasonic image classification using UWPI images. The authors compared conventional image processing methods and gaged the performance of these methods using the latest convolutional neural network (CNN) approach. As a result, their proposed method can achieve the highest accuracy whether or not there is a flaw on a plate. Moreover, their study provided new insight into the use of wavefield images and DL algorithms for automatically detecting defects in plate-like structures. However, the aforementioned research only solved the binary classification problem to detect whether the damage in an aluminum plate existed or not. In our research, however, we applied CNN for the quantification problem to determine the looseness in the bolts.

In a recently published article from our research team, Tola et al. [45] dealt with the monitoring of bolt joints by applying an ultrasonic wavefield energy analysis using an Nd:YAG pulsed laser scanning system. Here, a pulsed laser was used to excite the structure under investigation, and the wavefield data were measured using an acoustic emission sensor. To obtain the full wavefield information, a laser was used to scan the measurement area and excite the structure at different points. According to the micro-contact theory, it was expected that bolts fastened with higher torque levels have a larger contact area. Therefore, the reflected wave energy will be smaller. This was exploited in the proposed approach, where the wavefield information was filtered in the wavenumber domain and the energy in a certain time and spatial window wass computed based on the continuous wavelet transform using a Morlet mother wavelet. This approach was tested on an aluminum box girder, where the bolt under investigation is fastened using different levels of torque. The results obtained confirmed the assumptions regarding the energy distribution and demonstrate that the approach can be used for the monitoring of bolt joints.

However, similar to the other conventional methods in the literature, this research did not consider an AI approach to deal with the problem, as it suffers from the problem of not being able to automate the system.Therefore, developing an approach for automatically quantifying the looseness in bolted joints by combining full-field ultrasonic and DL algorithms is currently unaddressed but a worthwhile topic of research.

Hence, the contributions of this research are as follows:

- Introduce a new perspective of bolt looseness quantification which utilizes a DL-based computer vision algorithms with full-field ultrasonic data.
- Determine the applicability of a deep convolutional neural network (DCNN) algorithm and full-field ultrasonic data to estimate the looseness in bolted joints.
- Compare the effects of signal processing techniques for full-field ultrasonic data according to the performance of DL algorithms.

In this study, a non-contact laser scanning system was used for the generation of ultrasonic waves, and a DCNN was used for the automatic estimation of looseness in the bolt joints. The procedure for the proposed approach is as follows: A Q-switched Nd:YAG pulsed laser and an acoustic emission sensor were used for excitation and detection of an ultrasonic signals, respectively. Then, a 3D full-field ultrasonic data set frame was generated using the UWPI system. Next, the focus was on proving the necessity of data augmentation techniques. A number of comparative studies were conducted on the raw full-field ultrasonic data.

Subsequently, several signal processing techniques were selected to construct a processed data set. Five data sets were created: D1, a raw wavefield; D2, filtered down-propagation wave data; D3, filtered up-propagation wave data; D4: standing wave data, and D5, wavenumber adaptive image filtered data. Eventually the DCNN algorithm was used to estimate the looseness of bolted joint. The proposed approach was also compared with k-nearest neighbor (kNN), support vector regression (SVR), and deep artificial neural network (DNN) approaches. Following the application of a CNN, gradient-weight class activation mapping (Grad-CAM) was conducted to examine the region in which the DL algorithm identified in the wavefield. The t-SNE approach was also used in a 2D visualization of the features of each effective data set. Consequently, the proposed approach shows significant potential for incorporating an automatic looseness estimation from laser-generated ultrasound and DL algorithms.

This paper begins by describing the theoretical background. The proposed method is then described step-by-step in Section 3. The experimental validation results are presented in Section 4. Finally, Section 5 presents the conclusions of our study.

## 2. Theoretical Background

To introduce the proposed method, first the theoretical backgrounds are presented in the following order. The micro contact theory is briefly described in Section 2.1, and UWPI and signal processing techniques for full-field ultrasonic data are then described in Section 2.2 and Section 2.3, respectively. Finally, Section 2.4 outlines the proposed DCNN model.

### 2.1. Micro Contact Theory

**Figure 1:**
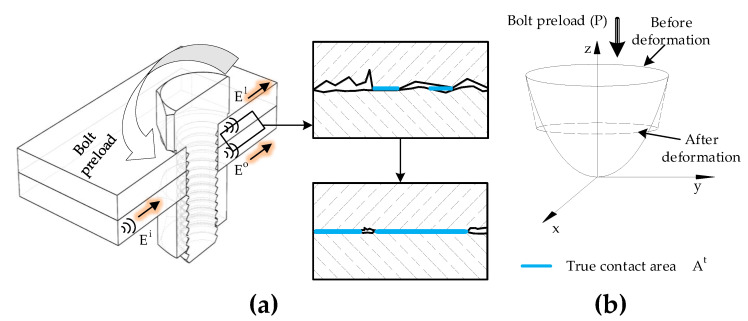
Changes of contact conditions at the bolted joint: (**a**) bolted joint prototype and microscopic view of the contact area, and (**b**) geometry of single asperity contact.

From a microscopic perspective, all machined surfaces are filled with a large number of asperities, and thus bolted joint surfaces can have partial contact with the underlying plate at their imperfect interface [48]. As illustrated in Figure 1a, if the preload of the bolt increases, the true contact pressure at the imperfect interface increases. However, it is understood that the actual contact area is less than the theoretical contact area [15]. Based on the models of the sinusoidal surface and the classic theory of Hertz contact [49], there exists a relationship between the true contact area $A^{t}$ and the pressure *P* (Figure 1b):

(1) $$A^{t}\propto C\sqrt{P}$$

where *C* is a constant. Furthermore, the wave energy dissipation phenomenon at the imperfect bolted joints can be quantified because waves are the means of energy transmission based on an analysis of wave propagation at the interface [50,51]. Moreover, based on the fact that first the wave is incident at the interface, before it goes through the interface some waves are reflected back while the rest goes into the interface. The wave portion that went into the interface again is divided into two, a portion is lost and the other portion passes through interface. With reference to Figure 1a, assuming that $E^{i}$ is the energy from incoming waves, after passing through the micro contact interface, the waves are split into lost waves and transmitted waves. Let $E^{l}$ be the lost wave’s energy and the energy transmitted be $E^{0}$. Based on previous research [48], the amounts of wave leakage and dissipation are known to be proportional to the actual contact area:

(2) $$E^{l}\propto A^{t}$$

The transmitted wave energy can be used as the tightness index for bolt-loosening detection. However, the increase in torque does not always lead to an increase in energy level under circumstances in which the assumptions of Equation (2) fail to hold. Moreover, if the torque reaches a certain level, the joint becomes somewhat saturated [52].

However, the amount of wastage of ultrasonic wave energy that is distributed through the bolted connection can be regarded as an index to assess the status of the bolted joints.

In addition, little research has been conducted on the use of ML or DL algorithms in the development of an automated looseness estimation system for bolted joints. Therefore, in this study, a DL algorithm, called DCNN is applied to examine the wave energy in the region around a bolted joint, and results are used to estimate the looseness.

### 2.2. Ultrasonic Wave Generation Mechanism Using Pulsed Laser: Scheme

**Figure 2:**
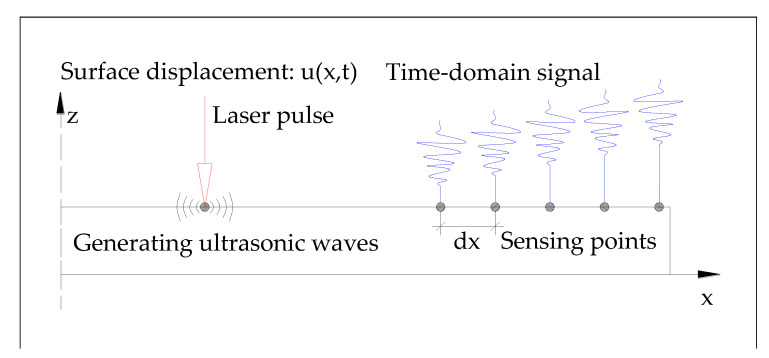
Ultrasonic wave mode generation in a plate. A source of impulsive pressure is applied to the surface and the resulting time records are tracked at different locations on the surface [53].

Figure 2 illustrates the ultrasonic wave generation via a pulsed laser [53]. When a pulsed laser beam impinges onto the target structure, a number of different physical phenomena can occur. The basic problems of the thermoelastic generation of ultrasound can be broken down into three sub-problems: medium absorption of the electromagnetic energy, reflection, and transmission of the laser radiation. As a result of these processes, the absorbed laser energy causes localized heating of the region, leading to thermoelastic expansion of the material and ultrasonic wave generation [31,53,54].

**Figure 3:**
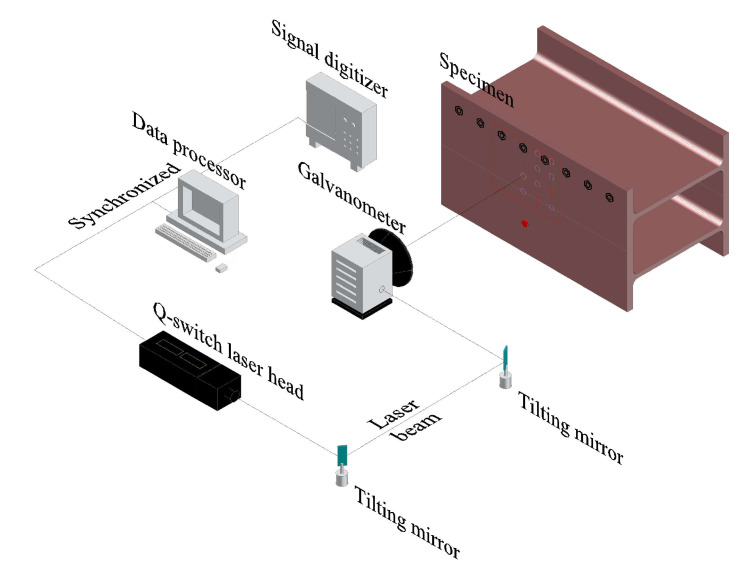
Conceptual diagram of an ultrasonic wave propagation imaging system.

Figure 3 shows the UWPI system setup used in previous studies [32,36]. This system includes a Q-switched laser system, a galvanometer-based laser mirror scanner, an ultrasonic sensor, a high-speed digitalizer, and an image processor. This is a static setup system with a distance of 1300 mm from the target structure. In this study, the Q-switched Nd:YAG diode-pumped solid-state laser had a wavelength of 532 nm and a maximum frequency of 20 Hz pulse repetition. The galvanometer had a maximum rotation speed of 100 rad/s and an allowable scanning angle of ±0.35 rad. The maximum energy of each pulse was 55 mJ, the diameter of the laser beam was 0.45 nm, and the energy density was 4.15 mJ/mm^2^.

Detailed information on the laser system is shown in Table 1.

**Figure 4:**
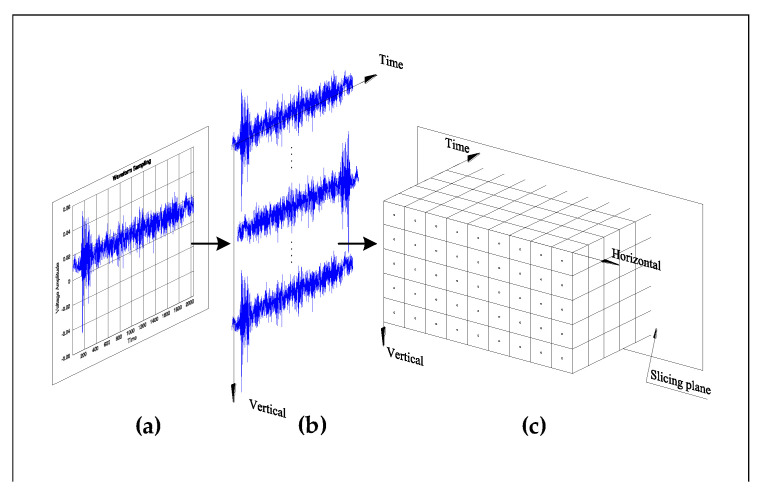
Process of ultrasonic wave propagation imaging (UWPI) system. (**a**) Measurement of time-domain response at each impinging point. (**b**) Placement of the measured signals at their corresponding laser impinging points that are outlined at the image processor. The result is a vertical plane containing all the impinging points and an into-the-page axis representing the time axis. (**c**) Slicing along the time axis of the 3D space.

The pulsed laser beam passes through the laser mirror scanner designed to operate the galvanometer at a wave length of 532 nm so that the beam is directed to a specific point on the target structure. The operating angles of the galvanometer’s two mirrors are orthogonal to each other, allowing the laser beam to quickly scan the 2D space. At the end of the laser scanner system, the f-theta lens mirror reflects the laser beam and focuses it onto the target scanning area. Ultrasonic wave signals could be assembled across the target surface by running the ultrasonic excitation on the structure vertically upward, stepping horizontally, and then scanning vertically downward. An acoustic emission (AE) sensor made of lead zirconate titanate piezoelectric ceramics was selected to measure the ultrasonic wave response. Ultimately, Figure 4 shows the process of the UWPI system and the three steps that were used to visualize the propagation of waves:

1. Measuring the time-domain response at each impinging point.
2. Placing the measured signals at their corresponding laser impinging points that are outlined at the image processor (resulting in a vertical plane containing all impinging points and an into-the-page axis representing the time axis).
3. Slicing along the time axis of 3D space.

Therefore, images of wave propagation can be obtained by slicing over time. In this respect, w(x,y,t) is the 3D data set containing information about a wavefield [32].

### 2.3. Full-Wavefield Signal Processing

The measured response data need further processing to obtain damage-sensitive features [55]. The wave images should be able to distinguish the difference between the intact and damaged cases [56]. The following signal processing techniques significantly affect the robustness of the performance of DL algorithms. If the function used is not associated with the damage, then the images of the wave in the damaged specimen have a complex representation; thus, DL algorithms must be used to meet the challenge of damage detection. On the other hand, if an appropriate signal statistic is chosen, then the results will improve.

It is extremely difficult to know exactly which signal processing technique is suitable for each type of damage. To the best our knowledge, there is only rare research in DL applications for full-field ultrasonic data. Thus, a sufficient number of techniques should be applied for a demonstration because there is no reference for validation; in this research, three different techniques [57] were utilized and five processed data sets were generated for comparison as described in the following subsections.

#### 2.3.1. Reflection Separation

The filtering process was first applied in research [58]. The step-by-step procedure is as shown in the following:

1. The filtering process starts with a 3D Fourier transformation that produces the wavenumber frequency domain representation of the wavefield $W\left(k_{x},k_{y},\omega \right)=F_{3D}\left\{w(x,y,t)\right\}$.
2. The elimination of waves in a wavenumber-frequency domain having a positive or negative sign at a fixed frequency filters a portion of the wave propagating in a specific direction $\bar{W}\left(k_{x},k_{y},\omega \right)=W\left(k_{x},k_{y},\omega \right)R\left(k_{x},k_{y},\omega \right)$.
3. Eventually, the filtered wavenumber-frequency’s inverse 3D Fourier transformation was used to extract the filtered data $\bar{w}\left(x,y,t\right)=F_{3D}^{-1}\left\{\bar{W}\left(k_{x},k_{y},\omega \right)\right\}$.

Here, $R(k_{x},k_{y},\omega )$ is the window function used for filtering and $R(k_{x},k_{y},\omega )$ is different for removing up-propagation waves $R_{up}$ and down-propagation waves $R_{down}$ as follows:

(3) $$R_{up}\left(k_{x},k_{y},\omega \right)=\left\{\begin{array}{c} 0,k_{x}\geq 0\\ 1,k_{x}<0 \end{array}\right\}$$

and

(4) $$R_{down}\left(k_{x},k_{y},\omega \right)=\left\{\begin{array}{c} 1,k_{x}\geq 0\\ 0,k_{x}<0 \end{array}\right\}$$

Both ${\bar{w}}_{up}\left(x,y,t\right)$ and ${\bar{w}}_{down}\left(x,y,t\right)$ are examined in the following sections.

#### 2.3.2. Isolated Cumulative Standing Wave Energy

As presented in [40], the cumulative standing wave energy (CSWE(x,y)) is calculated as follows:

(5) $$CSWE\left(x,y\right)=\int_{t_{1}}^{t_{2}}\left(w^{2}\left(x,y,t\right)-{\bar{w}}_{up}^{2}\left(x,y,t\right)-{\bar{w}}_{down}^{2}\left(x,y,t\right)\right)dt$$

#### 2.3.3. Wavenumber Adaptive Image Filtering

The wavenumber adaptive image filtering technique was implemented in a previous research [59,60]. Similar to the previous technique, the 2D window function $M(k_{x},k_{y})$ plays as a significant role in filtering the unwanted signals.

(6) $$\bar{W}\left(k_{x},k_{y},\omega \right)=W\left(k_{x},k_{y},\omega \right)M\left(k_{x},k_{y},\omega \right)$$

The filter mask is as follows:

(7) $$M\left(k_{x},k_{y},\omega \right)=\left\{\begin{array}{c} 0,W_{AVG}(k_{x},k_{y}\geq threshold\\ 1,otherwise \end{array}\right\}$$

According to [61], the threshold can be set at approximately 2% of the averaged wavefield in the wavenumber domain $W_{AVG}(k_{x},k_{y}$) points with the highest values. Therefore, five different data sets were created as follows:

- D1: Raw full-field ultrasonic data;
- D2: Filtered down-propagation wave data;
- D3: Filtered up-propagation wave data;
- D4: Standing waves data;
- D5: Wavenumber adaptive image-filtered data.

### 2.4. Deep Regression Convolutional Neural Network

Convolutional networks, also known as convolutional neural networks (CNNs), were first introduced by LeCun et al. [62]. CNNs help computers learn and understand a structure based on a hierarchy of concepts by collecting information from training data. A typical CNN architecture includes various types of processing layers, including a convolution, batch normalization, pooling and fully connected layers. Detailed explanations of these terms can be found in [63].

In this study, a DCNN was applied for a regression problem. First, given the target data pair $\left(x_{i},c_{i}\right)$ with $X=\left\{x_{1},\ldots x_{n}\right\}$ and $C=\left\{c_{1},\ldots c_{n}\right\}$, where $x_{i}$ is an input full-field ultrasonic data, $c_{i}$ is the corresponding torque value. The final goal was to estimate the unknown torque value c* for the new input wavefield data x*.

Suppose *W* is an unknown network weight, $f(W;x_{i})$ is then the prediction of the network, and *l* is a loss function. Using an optimization algorithm, such as RMSprop, Adadelta, Adam or Adamgrad, the optimization problem $minimize_{w}\sum_{i=1}^{n}l(f\left(W;x_{i}\right),c_{i}$ can be solved, and the weight *W* can be obtained.

**Figure 5:**
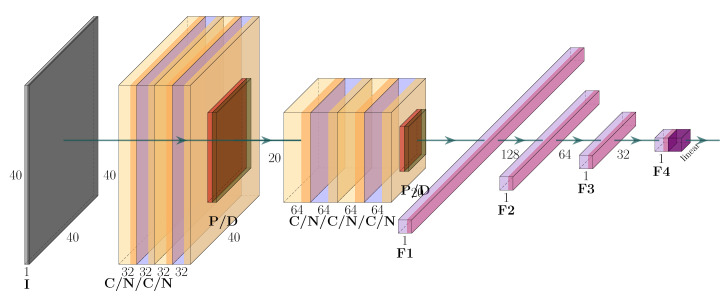
Deep-regression CNN architecture. I, input data; C, convolution layer; N, batch normalization layer; P, max pooling layer; D, dropout; F, fully-connected layer.

As shown in Figure 5, the CNN architecture used in this research was inspired from VGG-16 architecture [64]. However, in contrast to VGG-16, the VGG-like network used here had only five convolutional layers, while all the kernel filter sizes were 3 × 3 (C). Following each convolutional layer is the batch normalization layer (N). Max pooling layers (P) were applied between these C blocks. In addition, after the second and fifth convolution layers, 0.25 dropout (D) was added. This set of convolutional layers was followed by three fully connected layers, with 128, 64, and 32 incoming connections using ReLU activation. Finally, to solve the regression problem, a fully connected layer with one incoming connection and a linear activation output was added.

However, given the success of a DL in different applications, the interpretability of black-box CNNs remains a topic of considerable research. Sevaraju et al. [65] first introduced a technique, called gradient-weight class activation mapping (Grad-CAM) in 2017. This technique makes any model based on CNN more clear by offering visual explanations. Grad-CAM uses gradient knowledge that flows into the CNN’s last convolutional layer to allocate an importance to each neuron for a particular interest decision. After obtaining a trained model, the Grad-CAM technique is applied to determine the region where CNN followed the decision-making process. Therefore, in this study, some filtering steps were used to improve the accuracy and demonstrate the correctness of this Grad-CAM assumption.

## 3. Proposed Method

**Figure 6:**
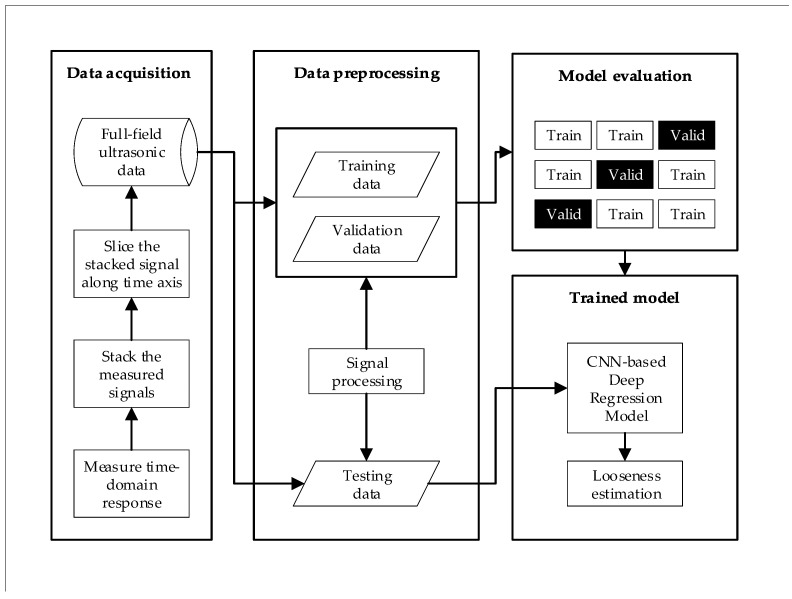
Proposed approach for estimating the looseness of the bolted joints using a deep convolutional neural network (DCNN).

Figure 6 demonstrates the process of the proposed method for estimating looseness in bolted joints. This can be summarized as follows:

1. First, the signals are measured at each impinging point using Nd:YAG pulsed laser scanning and an R-CAST AE sensor. The UWPI process is then performed, after which the full-field ultrasonic data sets are produced.
2. The second phase starts with the application of signal processing techniques for full-field ultrasonic raw data.
3. A model evaluation process is utilized for choosing the best model performance on different data sets.
4. Finally, the DCNN model is generated to estimate the looseness value of bolted joints after training and optimization.

## 4. Performance of Proposed Approach

### 4.1. Experiment Setup

**Figure 7:**
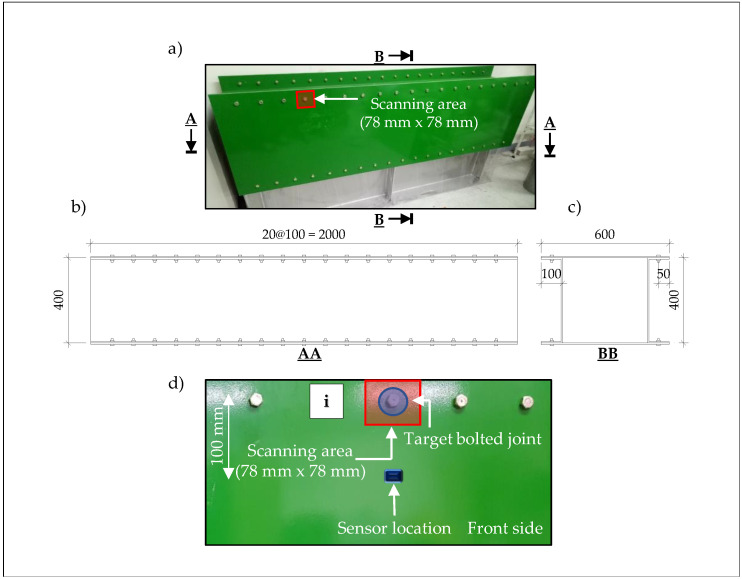
The 6061-aluminum box girder specimen: (**a**) front view with scanning area size, (**b**) AA cross-section, (**c**) BB cross-section, (**d**) sensor location setup. Here, i represents the bolt number being scanned, i = 1:25.

The bolt connections of a 6061-aluminum box girder were used to assess the feasibility of the proposed approach. Figure 7a–c show images and drawings of the sample box girder, respectively. A digital torque wrench, model BEM-60S3, with maximum capacity of 60 Nm, and a minimum of 3 Nm was used to create an artificial bolt looseness condition.

In this study, 25 bolted joints were scanned. The first 20 bolts were torqued in equal 5 Nm increments from 5 Nm to 25 Nm, and were used to perform experiments for training. The last five bolts with continuous torque values from 3 to 25 Nm were chosen for testing the model. One of the main reasons these values were chosen was that the torque limit was based on the bolted joint diameter, and it was challenging to control the torque below 3 Nm.

Referring to the UWPI process, the scanned area inside which was the target bolt connection was 78 × 78 mm. This system created a 40 × 40 point grid (total = 1600 points) with a point-to-point interval of 2 mm. That is, the sampling resolution for the space was 2 mm. In addition, with our GUI-based LabView VI. for data acquisition, the scanning process was carried out easily by using a for-loop to adjust the coordinates, as the distance between 2 bolts was set at 100 mm. The scanning process was completed within 1.5 min during each scanning experiment.

**Figure 8:**
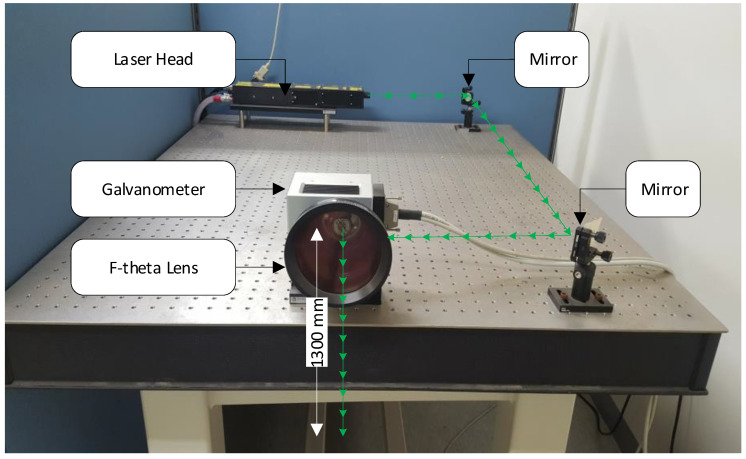
Experimental setup: A non-contact laser ultrasonic scanning system composed of a Q-switched Nd:YAG pulsed laser with a galvanometer for ultrasonic excitation scanning.

The parameters of the UWPI system used for the bolt connection inspection were introduced in the previous section. Figure 8 displays the overall test configuration. The distance from the galvanometer to the test specimen was 1300 mm. As shown in Figure 7d, an R-CAST AE sensor was mounted in a central position on the front side of the scanned surface to assess the multiple wave signals. Broadband AE sensor with lower and higher cutoff frequencies of 100 kHz and 2 MHz, respectively, were used. The resonant frequency of the sensor was 200 kHz ± 20%, and the maximum sensitivity at the resonant frequency was 120±3 dB.

The DL applications were conducted after the acquisition of all full-field ultrasonic data. The experiment was performed using an NVIDIA GeForce GTX 2080 Ti with 11 GB onboard memory and a DL framework [66] on an Intel Xeon CPU E3-1230 v6 with 32 GB.

### 4.2. Deep Regression CNN for Full-Field Ultrasonic Data

In this section, the implementation of the proposed approach is described. First, the input data in Section 4.2.1 is examined. Next, the process of the model evaluation and comparative studies are presented in Section 4.2.2. Eventually, the testing data are predicted with the optimal model in the last section.

#### 4.2.1. Input Data

**Figure 9:**
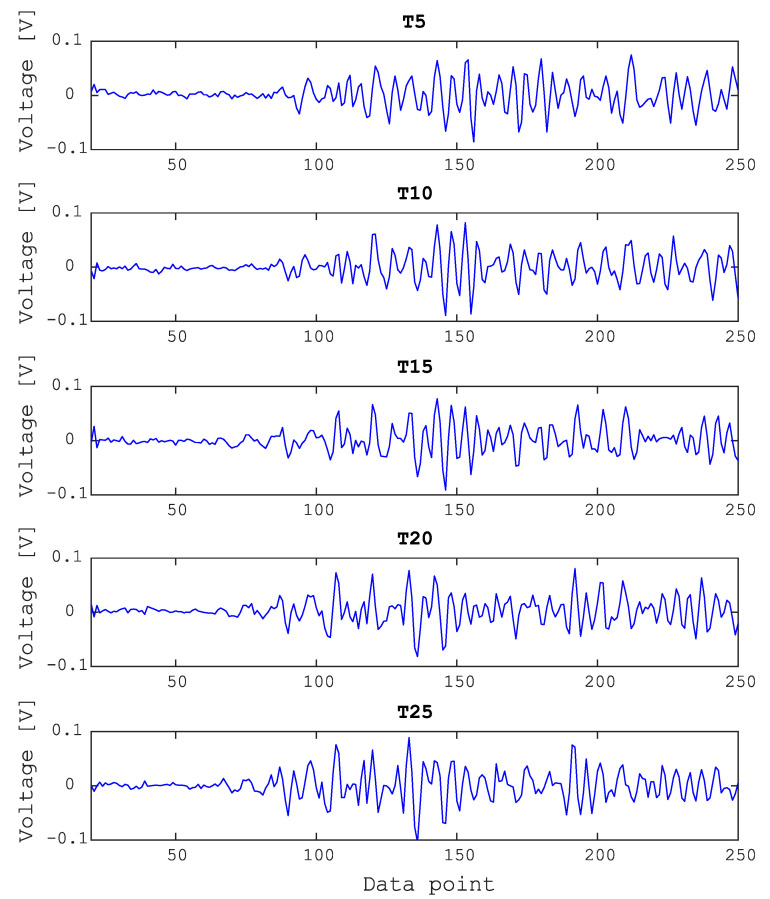
Typical waveform comparison between sample signals for torque values (T) from 5 to 25 Nm.

Figure 9 shows the typical wave forms of bolted joints, corresponding to a signal measured from five different torque values, 5 to 25 Nm in 5 Nm increments. To compare the differences between these cases, the sample signals are shown from the point at the top center of the scan area. Typically, the pulsed laser generates waves containing different frequency components, but as shown in Figure 9, the original signals already had a low noise level, and thus further processing was not required [33].

**Figure 10:**
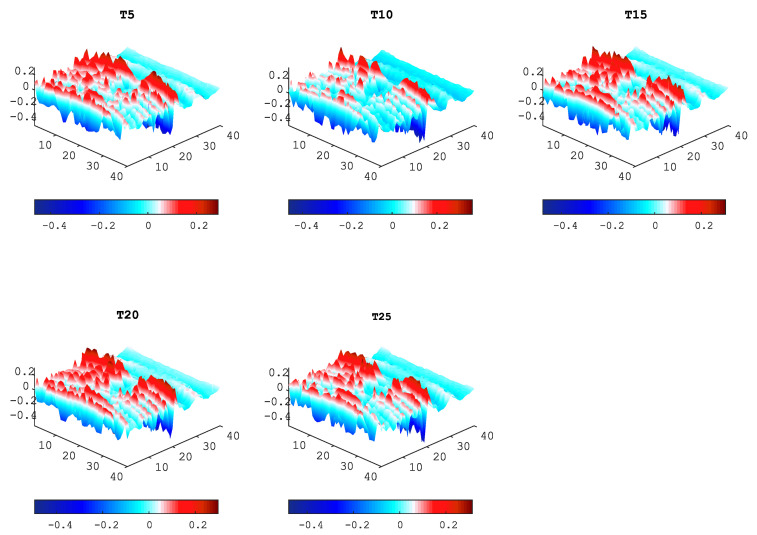
UWPI snapshots from five looseness (T) conditions at t=37μs.

Using the UWPI process, w(x,y,t) was obtained from all the specimen scanning points. Figure 10 shows snapshots of w(x,y,t) from five looseness conditions at 37μs. Nevertheless, it can be seen that the looseness of the bolt connection is difficult to characterize by a simple visualization if only the wave propagation video is used.

**Figure 11:**
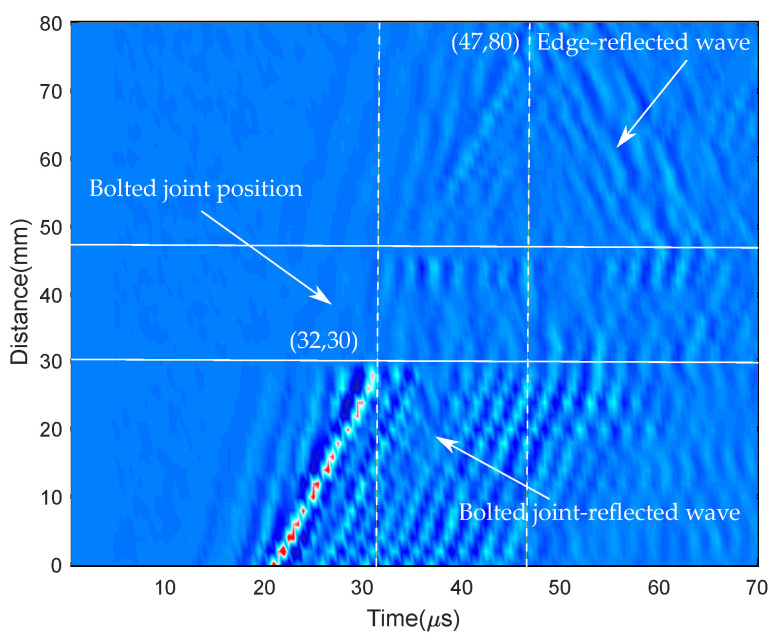
Cross-sectional image of the amplitude of the Lamb wave propagating through the target bolt.

To this point, raw full-field ultrasonic data were created. After that, the temporal margin describing the effective looseness in the bolted joints was identified based on several methods presented in our previous research [45]. However, for simplicity of the proposed method, here the temporal margin described by the time–distance–wave amplitude graph was chosen. Another automatic approach was presented in [45]. From this typical sample shown in Figure 11, it can be observed that the time when the wave front with a significantly high energy reached the target bolt was 32 μs, and the time when the wave reflection from the top edge emerged was around 47 μs. From these results, the temporal period 32 to 47 μs was utilized for the DCNN as the input data. In other words, 31 samples were extracted for each time experiment because the sampling frequency was $2.10^{6}$ sample/sec.

Specifically, w(x,y,t) was the sample input for the DCNN with a size of 40 × 40. The data set was then normalized using the rescaling method.

#### 4.2.2. Process of Model Evaluation

**Figure 12:**
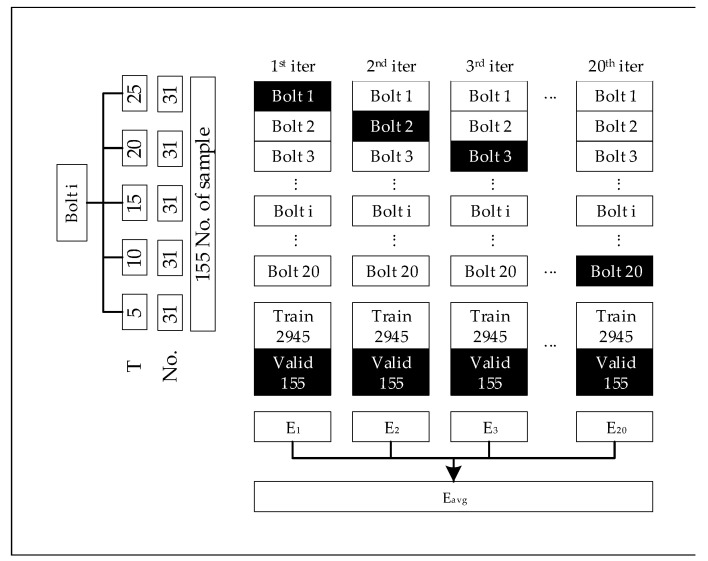
Model evaluation process. Each bolt contained 155 samples from five different torque values (T, torque, No., number of samples). Twenty iterations corresponding to 20 models were used for training, and the validation was on 19 bolts and 1 bolt, respectively. E, Error at each model; Eavg, averaged error used for model evaluation.

The aim here was to measure the effect of each data set on the performance of the DCNN. A cross-validation-like approach was conducted, as shown in Figure 12. As stated earlier, 20 bolted joints with 5 different torque values were observed. Among these, 20 iterations corresponding to 20 models were trained and validated on 19 bolts and 1 bolt, respectively. Alternatively stated, at each iteration, 2945 samples with a size of 40 × 40 were used to train and the validation was conducted on 155 samples.

The mean squared error (MSE), mean absolute error (MAE), and R2 score were considered for calculating the accuracy of the proposed approach. The MAE corresponds to the expected value of the absolute error loss, and the MSE is a risk metric equivalent to the expected square error or loss value. In the DCNN training phase, the MSE is used as a loss function and the MAE is used as a metric to validate the validation data set.

Assume that $\hat{c_{i}}$ is the estimated torque value of the *i*-th sample, and $c_{i}$ is the corresponding true value. Thus, the MAE and MSE estimated over N samples are defined as in Equations (8) and (9):

(8) $$MAE\left(c,\hat{c}\right)=\left(1/N\right)\sum_{i=0}^{N-1}\left|c_{i}-{\hat{c}}_{i}\right|$$

(9) $$MSE\left(c,\hat{c}\right)=\left(1/N\right)\sum_{i=0}^{N-1}{\left(c_{i}-{\hat{c}}_{i}\right)}^{2}$$

Equation (10) shows the function used for computing the coefficient of determination. This provides an indicator of the goodness of fit and thus is a measure of how well the model is likely to predict unknown samples, based on the ratio of the stated variance. Here, $\bar{c}$ is the mean of N sample torque values.

(10) $$R2\left(c,\hat{c}\right)=1-\left(\sum_{i=1}^{N}{\left(c_{i}-{\hat{c}}_{i}\right)}^{2}/\sum_{i=1}^{N}{\left(c_{i}-{\bar{c}}_{i}\right)}^{2}\right)$$

**Figure 13:**
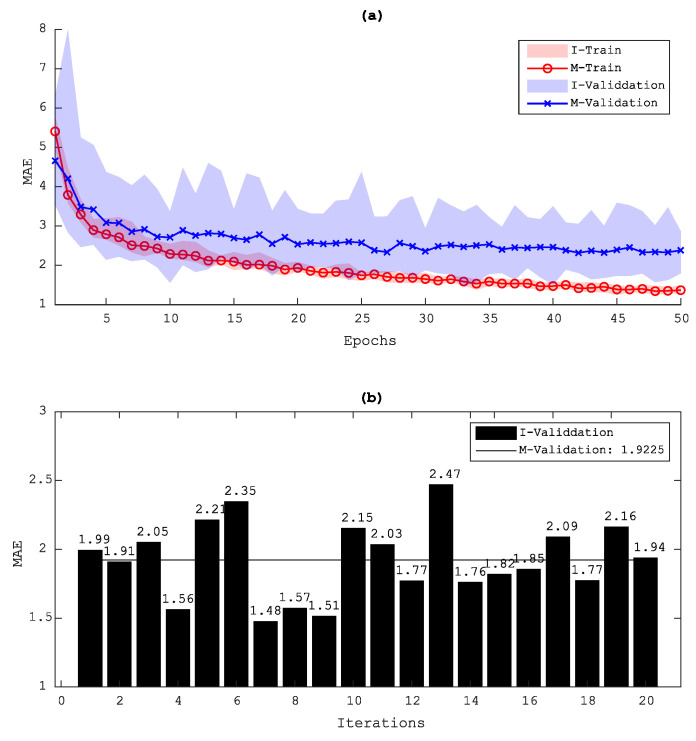
Evolution of the validation loss (MAE) during training for the D1 data set without using data augmentation: (**a**) validation loss and (**b**) minimum MAE of each model and the averaged result; I, iteration model; M, Mean results of the I model.

**Figure 14:**
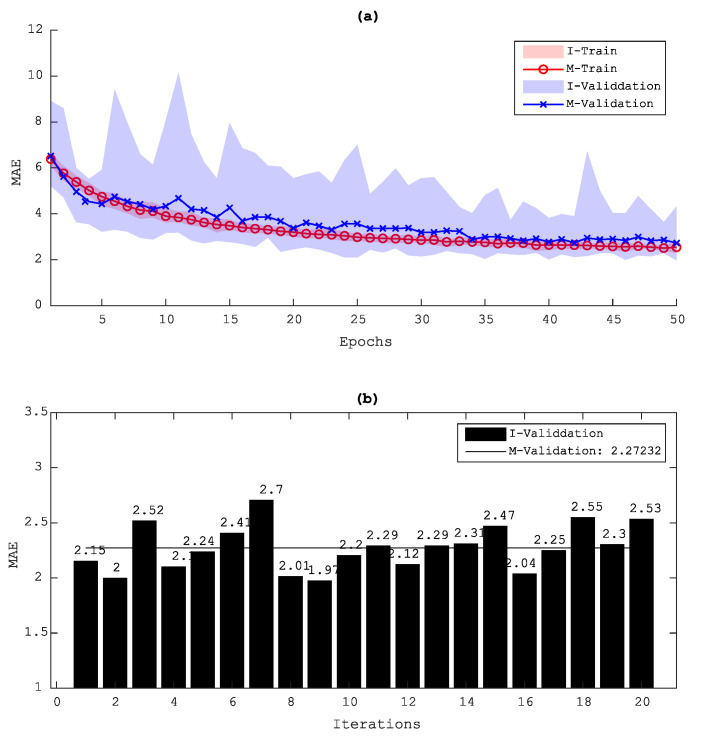
Evolution of the validation loss (MAE) during training for the D1 data set using data augmentation: (**a**) validation loss and (**b**) minimum MAE of each model and the averaged result; I, iteration model; M, Mean results of the I model.

Data augmentation is one of the most well-known techniques when training DL algorithms with small data sets. Data augmentation plays an important role to prevent the model from overfitting and also to improve its performance. However, depending on the complexity of each data set, this technique may give a poor result for data with clear patterns. Therefore, to decide whether or not to apply data augmentation in the data set, a comparison study was conducted between the results with and without such an augmentation. The data augmentation technique then augments the data by slightly rotating and randomly translating the data both vertically and horizontally. Figure 13 and Figure 14 show the loss evolution of both cases. As can be seen here, an overfitting occurred in the results without a data augmentation, and thus a data augmentation was applied for processing this type of full-field ultrasonic data.

**Figure 15:**
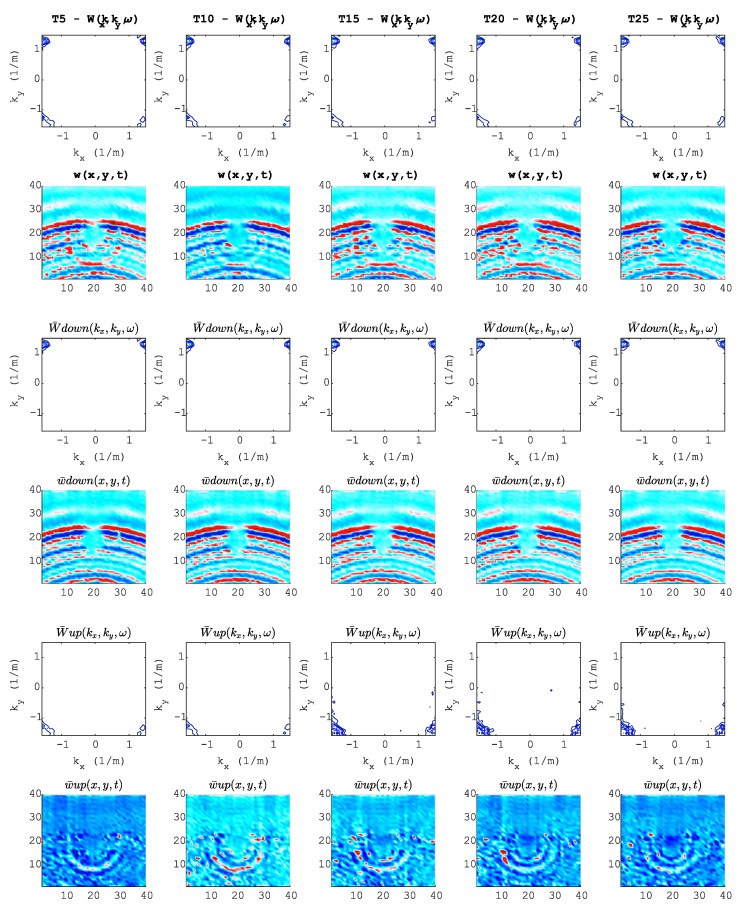
Preprocessing pipeline: Each column represents each torque value (T). Each row represents each processing step at t=37μs. Row 1: 3D FT before filtering; Row 2, full wavefield before filtering; Row 3, 3D FT after filtering down-propagation waves; Row 4, full wavefield after filtering down-propagation waves; Row 5, 3D FT after filtering up-propagation waves; Row 6, full wavefield after filtering up-propagation waves.

At this point, the network optimizer and training data augmentation technique are already suitable choices. As explained in the previous section, the signal processing techniques may have a better impact on the data set for DL algorithms. Figure 15 shows a preprocessing pipeline visualization of the selected wavefield at t=37μs and how five data sets were generated. The first and second rows show the full wavefield image in the wavenumber and space domains, respectively. In the third to sixth rows, a reflection separation is applied. Therefore, rows 2, 4 and 6 represent the D1, D2 and D3 sample data sets, respectively.

**Figure 16:**
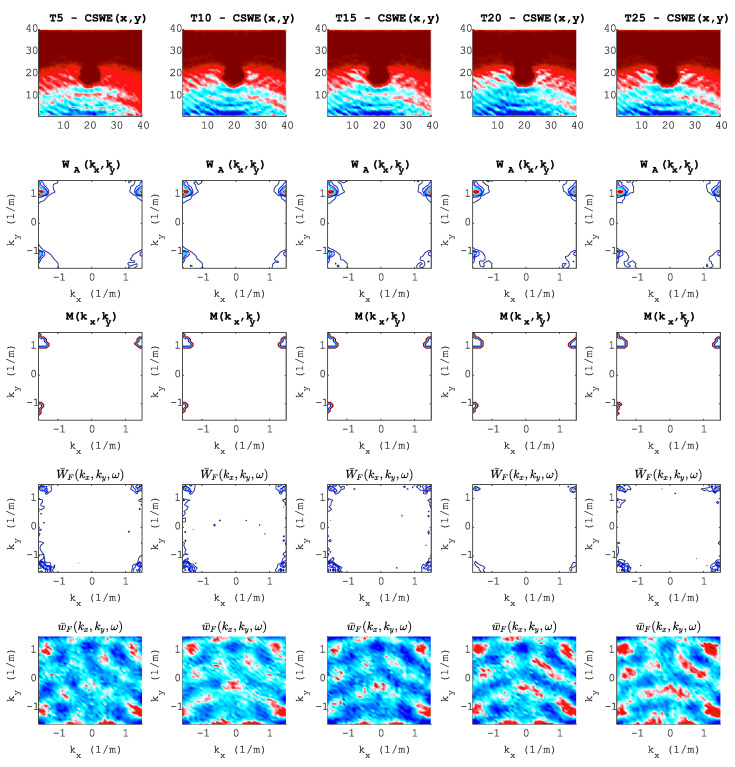
Preprocessing pipeline: Each column represents each torque value (T). Each row represents each processing step at t=37μs. Row 1, wavefield image of a standing wave; Row 2, averaged wavefield image in the wavenumber domain; Row 3, adaptive filter mask; Row 4, filtered wavefield image in the wavenumber domain; Row 5, wavefield image in space domain after adaptive filtering.

The first row of Figure 16 is the wavefield image of a standing wave (D4), and the second through last rows describe the steps of the wavenumber adaptive image filtering technique. This last signal processing technique starts by calculating the averaged wavefield in the wavenumber domain (second row) and then choosing the filter mask (third row). The fourth row shows the filtered wavefield image in the wavenumber domain, and the last row shows the filtered image in the space domain (D5).

Based on these five different data sets, the proposed approach using a DCNN was applied, and the results were then compared with a kNN, an SVR, and a DNN. To achieve a meaningful comparative study between these algorithms, several parameters were taken into consideration and a looping was conducted. Especially, the deep regression model using neural network (DNN) was developed carefully with deep layers and suitable hyper-parameters. In this analysis, k∈1,5,10,50,100 was used for kNN and C∈1,10,50, $\epsilon \in 10^{-2},10^{-1},$ was used to find the optimal results for SVR. DNN for the regression model was generated with 1600 input variables, together with three hidden layers of 128, 64, and 32 neurons, respectively. The linear activation function was finally used to process the output, while Adam was used as a network optimizer.

**Figure 17:**
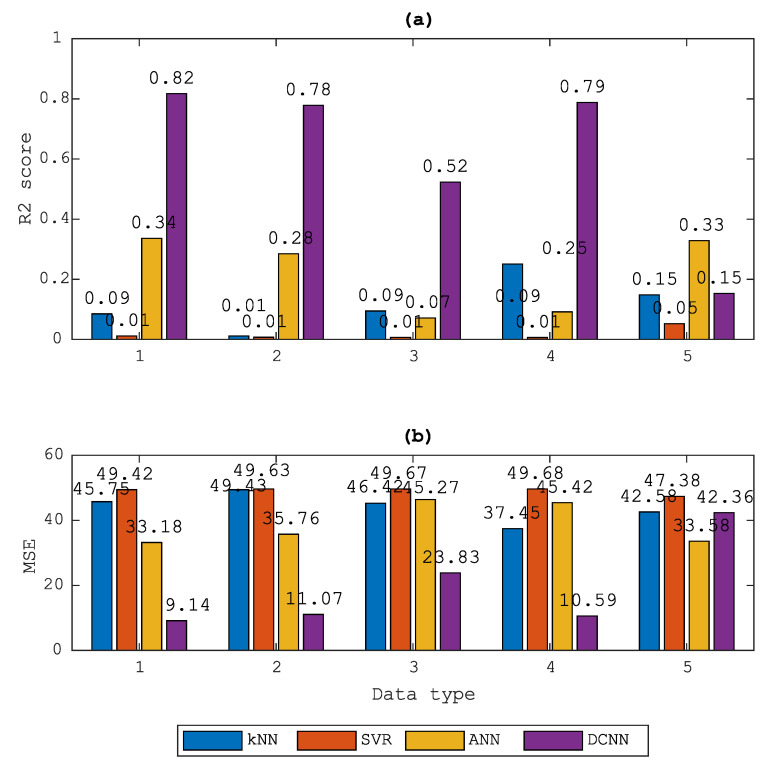
Comparison of accuracy in terms of R2 (a) and MSE (b) score from four different regression models: kNN, SVR, ANN and DCNN.

From the results shown in Figure 17, the proposed DCNN showed its effectiveness in almost every data set, especially in the raw (D1), the filtered down-propagation wave data (D2) and the standing wave data (D4). However, good results in the validation or development set are not a guarantee of good results for the test set. Therefore, to examine how well the DCNN model and signal processing techniques perform, the testing case was conducted.

#### 4.2.3. Model Prediction

As shown in Table 2, for bolt 1 and 2, similar torque values as in the training data set were tested. For bolts 3 to 5, to further ensure that the model can work well in the other looseness condition, a series experiment on different torque values, extending from 3 to 25 Nm, were conducted.

**Figure 18:**
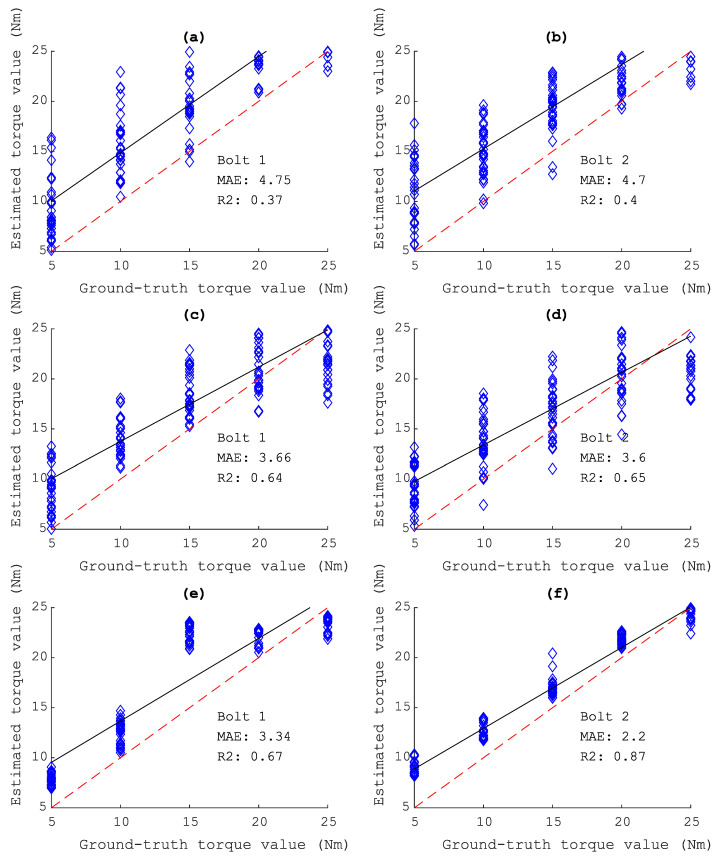
Estimated looseness using trained models. (**a**,**b**) Predicted results from the trained model using D1 data set on bolt 1 and bolt 2. (**c**,**d**) and (**e**,**f**) using D2, D4 data set, respectively.

As can be seen from Figure 18, the estimated torque is the mean of all iteration trained models. It is clear that by using the standing wave data set (D4), the DCNN can produce well estimated results, for bolt 1 with MAE=3.4, R2=0.67 and bolt 2 with MAE=2.2 and R2=0.87.

**Figure 19:**
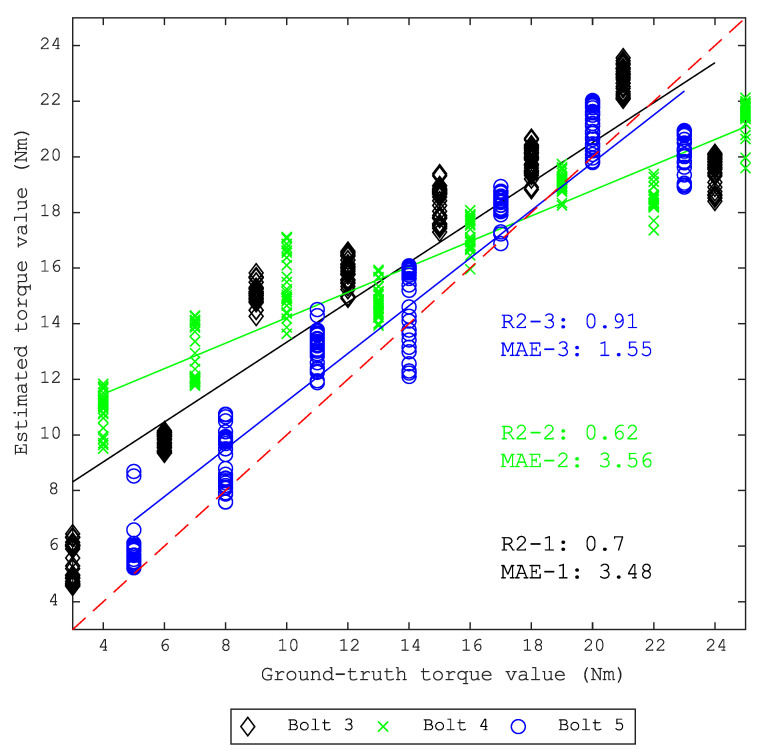
Estimated looseness using trained models on the D4 data set from bolts 3, 4 and 5.

Based on these results, the difficult test set with bolt 3, 4 and 5 continues to be used and the output is shown in Table 3. At this point, the D4, which uses standing waves data, outperforms other signal processing techniques in both metrics MAE and R2. Figure 19 shows the estimated torque value using DCNN trained models on the D4 data set.

**Figure 20:**
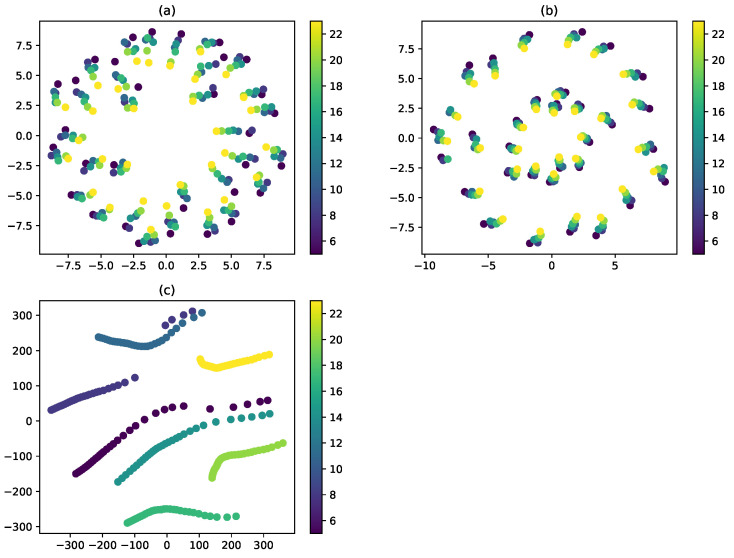
tSNE graph for seven different torque conditions on bolt 5 using different data sets. (**a**) D1, (**b**) D2, and (**c**) D4.

**Figure 21:**
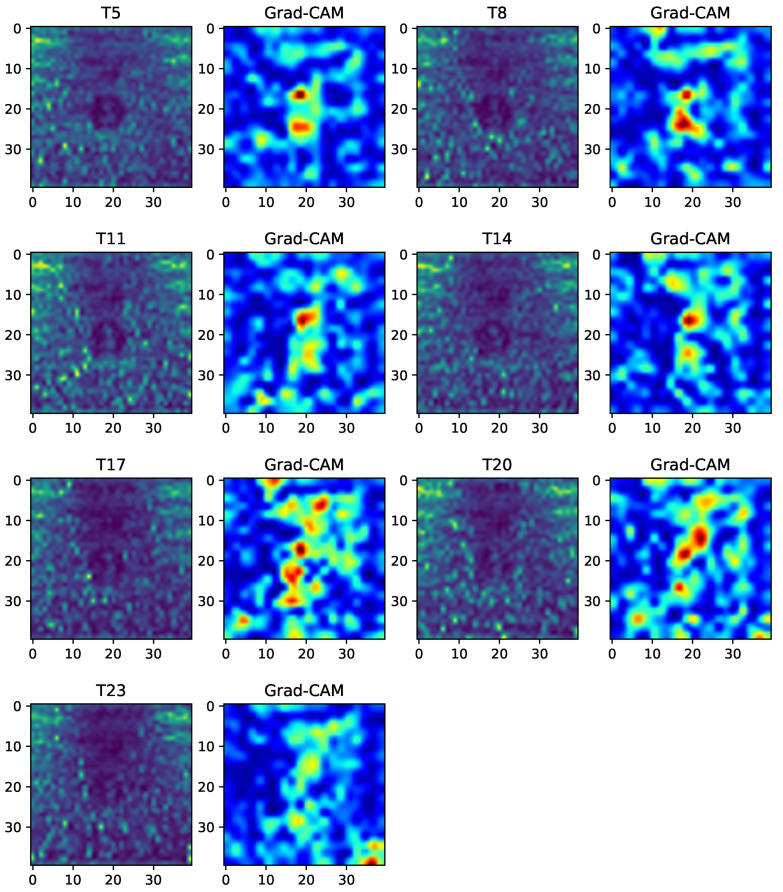
Grad-CAM images extracted from the D4 data set at time t=37μs.

One of the reasons, why the DCNN achieved better results on the D4 data set can be explained by using tSNE. Figure 20 shows the t-SNE 2D visualization from the D1, D2, and D4 data sets. As can be seen here, the t-SNE on D4 produced a clear cluster, more so than on D1 and D2. Thus, the DCNN made the decision-making easier, and Grad-CAM provided insight into the way the trained DCNN model supported the decision. Figure 21 demonstrates that, under each separate torque condition, the DCNN model emphasized the region around the bolt head. These results validated our assumption regarding the relation between ultrasonic energy and looseness according to micro-contact theory.

## 5. Conclusions

Looseness monitoring of bolted joints is necessary to maintain structural health. This study introduced the applicability of DL algorithms and non-contact laser scanning in the estimation of the looseness. First, a UWPI system, including a Q-switched Nd:YAG pulsed laser and an acoustic emission sensor, was employed to create frames of full-field ultrasonic 3D data sets. Then, a DCNN and several signal processing techniques were applied to obtain a better image of the DL performance. The results were also compared with conventional regression models, kNN, SVR, and DNN. The proposed solution showed a significant potential for an automated looseness estimation by combining laser-generated ultrasound data and DL. For bolt heads with sizes larger than M10, the proposed method can still be used for monitoring because the underlying theory remains the same. In addition, it can also be seen as a promising approach for other defect detection applications.

The main results are summarized as follows:

- Application of the data augmentation technique was necessary for the DCNN to produce acceptable results on full-field ultrasonic data.
- To obtain better results, an isolated cumulative standing wave energy can be used as a signal processing technique. In this research, the DCNN and this signal processing technique produced the highest R2 score and lowest MSE score, 0.91 and 1.55, respectively.

Nevertheless, there is still room for improvement of our method, which will be addressed in a future study:

- The UWPI system is still partially non-contact laser scanning, that is, the AE sensor is used as an ultrasonic receiver in the experiment and needs to be set up manually. To overcome this limitation, the LDV will be developed to replace the AE sensor for acquiring ultrasonic signals. Finally, the system can become fully non contact scanning and work without the need for setting up manually.
- The proposed method can be applied to the bolt in the straight line area only. In the cases of complex areas where the bolt locations are not on a straight line, the system will meet the challenge to excite and receive the ultrasonic signal. Thus, the method needs to improve the hardware of the device used for scanning.

## Figures and Tables

**Table 1:** Specifications of the laser system.

Laser Head	Galvanometer
Wavelength: 532 nm	Wavelength: 532 nm
Maximum laser energy per pulse: 55 mJ	Tracking error: 0.16 ms
Pulse repetition rate: 20 Hz	Positioning speed: 10 m/s
Pulse duration: 6.5 ns	Max. angular velocity: 100 rad/s
Beam diameter: 3 mm	

**Table 2:** Description of test bolts.

Tested Bolt	Torque Values (Nm)
1, 2	5, 10, 15, 20, 25
3	3, 6, 9, 12, 15, 18, 21, 24
4	4, 7, 10, 13, 16, 19, 22, 25
5	5, 8, 11, 14, 17, 20, 23

**Table 3:** Torque value prediction accuracy of the different data sets.

	D1		D2		D4	
	MAE	R2	MAE	R2	**MAE**	**R2**
Bolt 3	3.51	0.58	3.16	0.67	**3.48**	**0.7**
Bolt 4	4.23	0.42	3.23	0.63	**3.56**	**0.62**
Bolt 5	3.06	0.44	4.29	0.22	**1.55**	**0.91**

## Abbreviations

The following abbreviations are used in this manuscript:

DL	Deep learning
ML	Machine learning
NDE	Non-destructive evaluation
SHM	Structural health monitoring
Nd:YAG	Neodymium-doped Yttrium Aluminum Garnet
UWPI	Ultrasonic Wave Propagation Imaging
DCNN	Deep convolutional neural network
AE	Acoustic emission
kNN	k-nearest neighbors
SVR	Support vector machine
DNN	Deep neural network
Grad-CAM	Gradient-weight class activation mapping
